# Thoracic Spontaneous Spinal Epidural Hematoma in a Young Adult: A Case Report

**DOI:** 10.7759/cureus.115476

**Published:** 2026-08-31

**Authors:** Omar El Dishish, Qing Hang Tan, Ho Yin Kevin Wong, Dinesh Kumar

**Affiliations:** 1 Orthopaedics, Changi General Hospital, Singapore, SGP; 2 Orthopaedic Surgery, Changi General Hospital, Singapore, SGP

**Keywords:** case report, spontaneous spinal epidural hematoma, spontaneous spinal epidural hematoma (sseh), thoracic spontaneous spinal epidural haematoma, young adult

## Abstract

Spontaneous spinal epidural hematoma (SSEH) is an uncommon condition that may present with acute back pain and neurological deficits. Prompt diagnosis and timely intervention are important to optimise neurological recovery.

A 21-year-old female presented with a 5-day history of back pain associated with right lower limb weakness, gait instability, and bilateral foot numbness. Clinical examination demonstrated reduced muscle power (4/5) in the right lower limb and an ataxic gait. Magnetic resonance imaging (MRI) revealed a posterior epidural hematoma extending from T6 to T8 with significant spinal cord compression and cord oedema. The patient underwent urgent T6-T8 decompressive laminectomy and evacuation of the hematoma. Intraoperatively, no vascular malformation was identified. Histopathological examination demonstrated an organizing hematoma without evidence of vasculitis or malignancy, and subsequent investigations excluded coagulopathy and autoimmune disorders. Postoperatively, the patient achieved complete neurological recovery with restoration of full muscle power. At one-year follow-up, she had returned to her premorbid functional status, and repeat MRI showed no recurrence, with only mild residual cord signal changes consistent with gliosis.

SSEH should be considered in patients presenting with acute back pain and neurological deficits, even in the absence of identifiable risk factors. Early recognition, MRI evaluation, and timely surgical decompression can result in favourable neurological outcomes.

## Introduction

Spontaneous spinal epidural hematoma (SSEH) is a rare condition characterised by bleeding into the spinal epidural space without preceding trauma or iatrogenic intervention. Although it accounts for less than 1% of spinal canal space-occupying lesions, it represents a neurosurgical emergency because progressive spinal cord compression may result in permanent neurological deficits if diagnosis and treatment are delayed [1]. 

SSEH most commonly affects middle-aged and older adults, and shows a male predominance [2]. Reported risk factors include anticoagulant therapy, bleeding disorders, vascular malformations, and pregnancy; however, some cases remain idiopathic. Patients typically present with acute back pain followed by neurological deficits, often mimicking other spinal pathologies. Magnetic resonance imaging (MRI) is the imaging modality of choice, and early surgical decompression is recommended for patients with neurological impairment [3,4]. 

We report a case of true idiopathic thoracic SSEH in a 21-year-old young adult without identifiable predisposing factors, highlighting the importance of considering this diagnosis even in young patients presenting with acute back pain and neurological deficits.

## Case presentation

A 21-year-old woman presented to our centre with a 5-day history of back pain associated with right lower limb weakness. In addition, her gait has become unsteady due to limb weakness and bilateral foot numbness. She denied any past medical history or trauma to her back. Physical examination shows she had muscle power of 4/5 for all key motor units of her right lower limb. Gait analysis revealed an ataxic gait with poor dynamic balance.

Routine laboratory investigations, including complete blood count, coagulation profile, renal function, liver function, and inflammatory markers, were within normal limits. Autoimmune screening, including antinuclear antibody (ANA) and antineutrophil cytoplasmic antibody (ANCA), was negative. MRI revealed a heterogeneous signal abnormality seen at the posterior epidural region of the T6-T8 level (6cm in length), which was suspicious of an epidural hematoma. The hematoma caused cord compression from mid T6 to T7/8 level with associated cord oedema (Figure 1).

**Figure 1:**
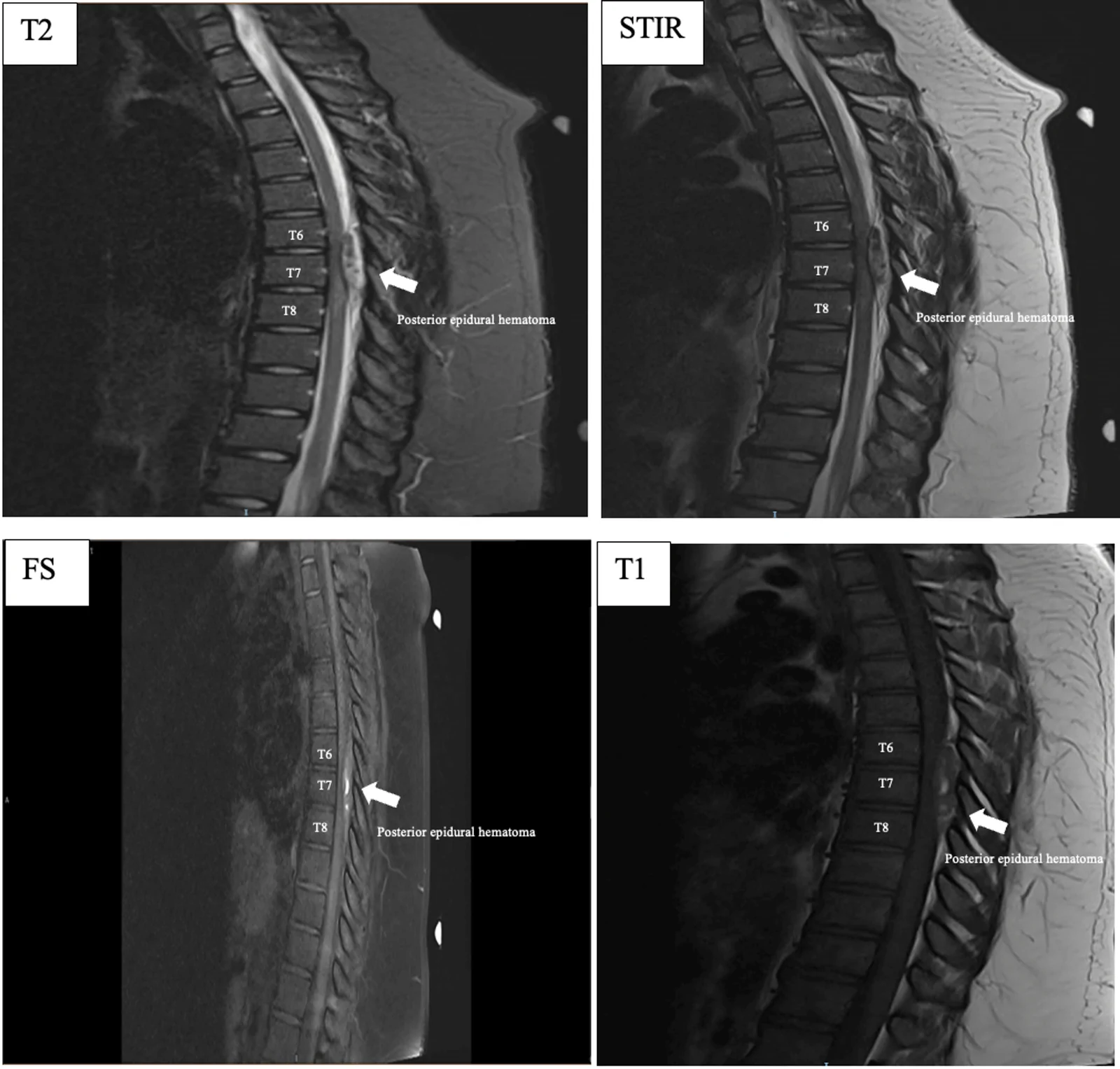
Sagittal view of preoperative magnetic resonance imaging (MRI) MRI demonstrating a posterior epidural hematoma (white arrows) extending from T6 to T8 with significant spinal cord compression and cord oedema. Top-left image: T2-weighted MRI; Top-right image: Short Tau Inversion Recovery (STIR) MRI; Bottom-left image: MRI fat-suppressed (FS) views; Bottom-right image: T1-weighted MRI

She underwent decompressive laminectomy from T6 to T8 with excision of the extradural lesion. Intraoperatively, no abnormal large extradural vessels were identified to suggest an arteriovenous malformation or dural arteriovenous fistula.

Histological examination demonstrated organizing blood clots with adjacent bone, marrow tissue, granulation tissue, and adipose tissue. Fibrinoid necrosis of the vessel wall was identified in a single medium-sized vessel; however, the remaining vessels showed no histological features of vasculitis (Figure 2). Overall, the histopathological findings were not considered diagnostic of vasculitis.

**Figure 2:**
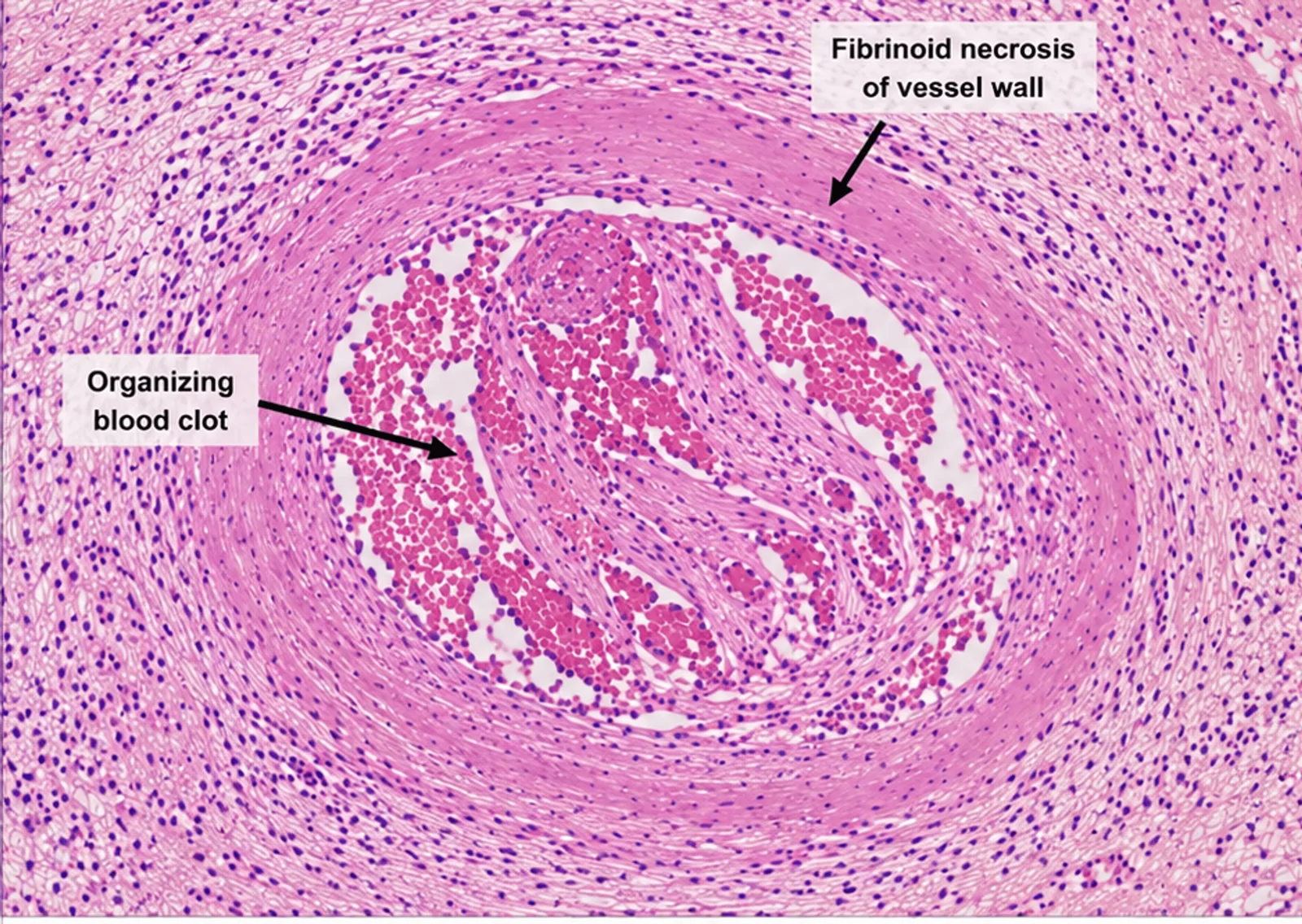
Intraoperative histological sections Histopathological examination (hematoxylin and eosin stain). Black arrows identify the organizing blood clot and a medium-sized vessel demonstrates focal fibrinoid necrosis of the vessel wall, whereas the remaining vessels show no histological features of vasculitis. Magnification: ×100 (overview) and ×400 (inset).

Postoperatively, she regained her muscle power of 5/5 in her Right lower limb. Given the histopathological findings, further investigations were performed to evaluate for underlying autoimmune disease and coagulopathy; however, all results were negative.

She underwent continuation of therapy with the rehabilitation team at our centre. Further investigations were performed to evaluate for underlying vasculitis. A rheumatology consultation was obtained, and based on the patient's clinical history, physical examination, and laboratory investigations, an autoimmune condition contributing to the SSEH was considered unlikely.

A repeat MRI performed approximately 1 year after the SSEH showed no residual or new epidural hematoma. However, persistent mildly increased STIR and T2-weighted signal intensity was noted within the spinal cord at the T7 level, suggestive of cord edema or gliosis (Figure 3). At the 1-year follow-up, the patient had returned to her pre-illness baseline, with full muscle strength in both lower limbs.

**Figure 3:**
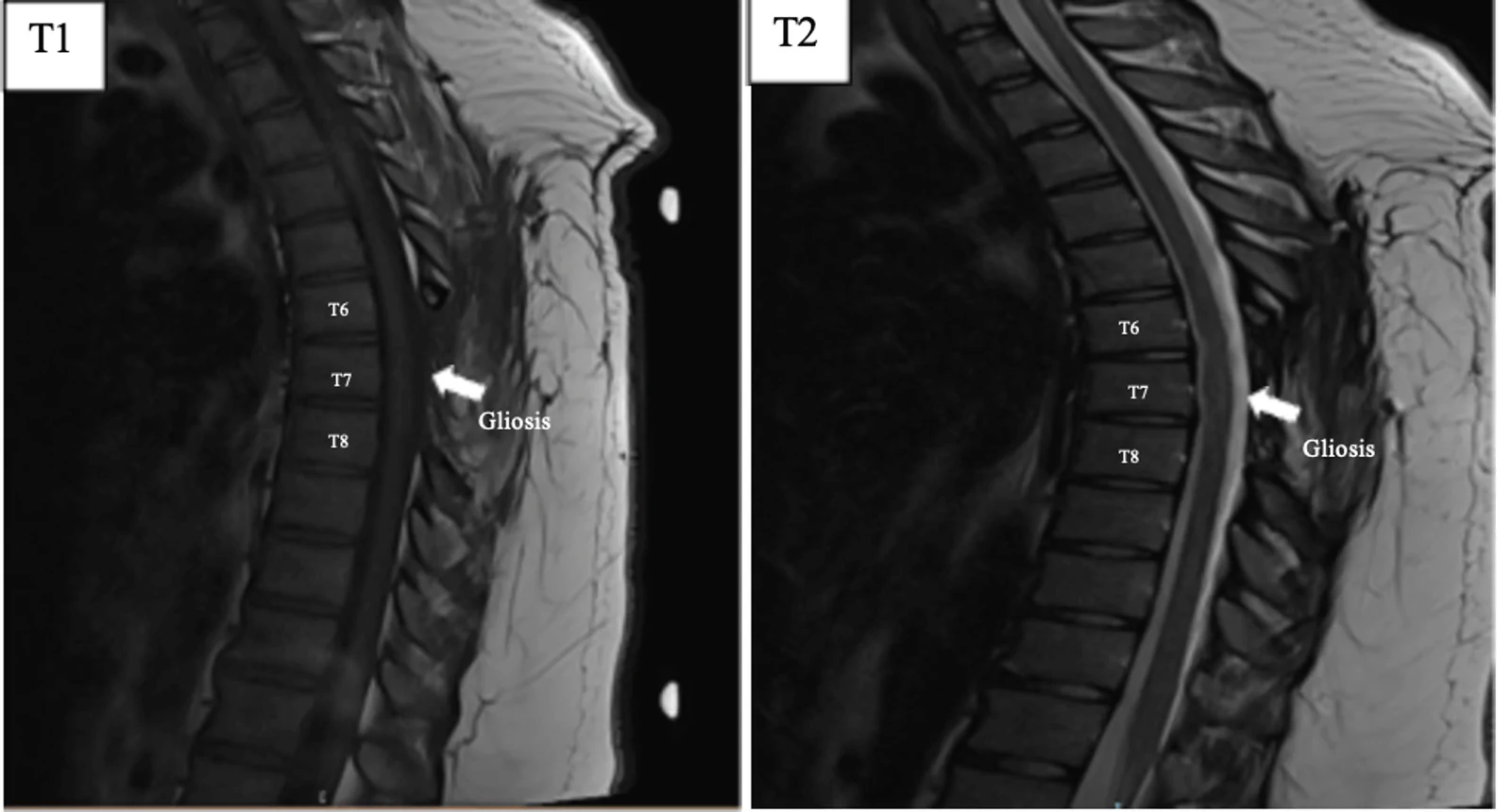
Sagittal view of the postoperative magnetic resonance imaging (MRI) of the thoracic spine one year after surgery Left: T1-weighted MRI; Right: T2-weighted MRI One-year follow-up MRI demonstrating complete resolution of the epidural hematoma with no evidence of recurrence. Arrow indicates mild residual T2/STIR hyperintensity at the T7 level, consistent with residual cord gliosis.

## Discussion

Documented cases of spinal epidural hematoma (SSEH) remain sparse, underscoring the clinical rarity of this condition. It is most frequently reported after the fourth or fifth decade. However, the presentation of this condition is sparsely documented among younger populations [2]. The present case involves a 21-year-old young adult, which is younger than the age group most commonly reported in the literature. This highlights that spontaneous spinal epidural hematoma can also occur in young adults despite its greater reported frequency in older individuals. In contrast to the established male predominance of this disease, the case presented herein features a female patient.

Although various precipitating factors for SSEH (anticoagulant therapy, pregnancy-related hormonal fluctuations, and haemophilia) have been identified, the etiology in this patient differs [5-7]. In the present case, no underlying cause for the hemorrhage was identified despite comprehensive evaluation. Laboratory investigations excluded coagulopathy and autoimmune disorders; intraoperative assessment showed no evidence of vascular malformation; and histopathological examination showed an organizing hematoma without evidence of vasculitis or malignancy. Taken together, these findings support the diagnosis of a true idiopathic spontaneous spinal epidural hematoma, highlighting the importance of considering this diagnosis even in young patients without identifiable risk factors. The source of bleeding could either be venous or arterial. Beatty and Winston et al. postulated that the bleeding is likely arterial in origin [8]. They further added that the epidural venous pressure is always lower than the intrathecal pressure; therefore, the presence of any bleeding would have been tamponade.

MRI has been the treatment modality of choice, as shown by many authors from their case reports [9,10]. The availability of MRI with a variety of imaging options (T1/T2/STIR/FS +C) in our centre helped in establishing the diagnosis in our case. Her presenting symptom of back pain, which is normally seen in cases of nerve root impingement, prompted us to get an MRI done. Initial scout imaging identified the lesion, which was subsequently characterized by a dedicated thoracic MRI.

Neurological outcome following SSEH is influenced by both the severity of the preoperative neurological deficit and the interval between symptom onset and surgical decompression. Although our patient presented five days after symptom onset with a relatively extensive thoracic epidural hematoma, the neurological deficit was limited to mild right lower limb weakness (Medical Research Council grade 4/5) without complete paralysis [11]. This relatively preserved neurological status may have contributed to the excellent postoperative recovery following urgent decompression. While earlier surgical intervention is generally associated with improved neurological outcomes, the present case illustrates that favourable recovery can still be achieved in selected patients with mild preoperative deficits despite delayed presentation [3,4]. As with our case report, evacuation of the hematoma surgically aided the patient's recovery. However, there are case reports of SSEH being treated conservatively, especially in the paediatric age group [12].

As this is a retrospective case report, some clinical parameters, including detailed neurological examination findings, standardized neurological outcome scores, and quantitative histopathological assessment of the number of vessels examined, were not documented and therefore could not be reported.

## Conclusions

Emergency medical personnel must include SSEH in the differential diagnosis for patients presenting with acute back pain, despite its rarity. Currently, MRI is the preferred diagnostic modality, and surgical intervention is indicated unless the patient exhibits a resolving neurological deficit.
